# The Prognostic Potential of circRNAs in Multiple Myeloma: Insights From Whole Bone Marrow and Purified Plasma Cells

**DOI:** 10.1111/jcmm.70215

**Published:** 2024-11-27

**Authors:** Theresa Jakobsen, Gro Grunnet Pløen, Alenka Djarmila Behsen, Holger Jon Møller, Torben Plesner, Karen Dybkær, Morten Nørgaard Andersen, Kristine Misund, Lasse Sommer Kristensen

**Affiliations:** ^1^ Department of Biomedicine Aarhus University Aarhus Denmark; ^2^ Department of Clinical and Molecular Medicine Norwegian University of Science and Technology Trondheim Norway; ^3^ Department of Clinical Medicine Aarhus University Aarhus Denmark; ^4^ Department of Clinical Biochemistry Aarhus University Hospital Aarhus Denmark; ^5^ Institute of Regional Health Science University of Southern Denmark Vejle Denmark; ^6^ Department of Internal Medicine, Section of Hematology, Lillebaelt Hospital University Hospital of Southern Denmark Vejle Denmark; ^7^ Department of Clinical Medicine Aalborg University Aalborg Denmark; ^8^ Department of Hematology, Clinical Cancer Research Center Aalborg University Hospital Aalborg Denmark; ^9^ Department of Molecular Medicine (MOMA) Aarhus University Hospital Aarhus Denmark; ^10^ Department of Hematology Aarhus University Hospital Aarhus Denmark; ^11^ Department of Medical Genetics St. Olavs Hospital Trondheim Norway

## Abstract

Multiple myeloma (MM) is a haematological malignancy with abnormal proliferation of plasma cells in the bone marrow (BM), and MM patients with highly proliferative plasma cells have reduced overall survival. Circular RNAs (circRNAs) are endogenous, non‐coding molecules that are promising biomarkers in cancer. Here, we present the largest study of circRNAs in MM to date and explore the prognostic potential of circRNAs and the link between proliferation and circRNA expression in MM. We performed deep total RNA sequencing (RNA‐seq) on two cohorts: one cohort consisting of 45 whole BM MM patient samples and 13 healthy controls (HCs), and another cohort consisting of 43 CD138‐purified plasma cell MM patient samples. We found that circRNAs are globally upregulated in the whole BM of MM patients compared to HCs. In whole BM, low proliferation and high circRNA levels were associated with a poor prognosis, while in purified plasma cells, low proliferation and high circRNA levels were associated with a favourable prognosis. Individual circRNAs from purified plasma cells were found to be significantly associated with MM patient outcomes and provide additional prognostic value to the proliferative indexes. Together, our findings emphasise the potential of circRNAs as prognostic biomarkers in MM.

## 1 Introduction

Multiple myeloma (MM) is the second most common haematological malignancy and accounts for 1% of all cancers [1]. Each year, 32,000 patients are diagnosed with MM in the United States alone [2]. The median age at diagnosis is 65 years [3], and the median overall survival is 5–8 years [4]. Despite significant development of new treatment options within the past 15 years, the vast majority of patients experience multiple disease relapses, and MM remains an incurable disease [4].

Normal bone marrow (BM) is a highly proliferative tissue [5], containing only 1%–2% plasma cells [6]. MM is defined by clonal expansion of plasma cells, and one of the diagnostic criteria is more than 10% clonal BM plasma cells [4]. MM disease progression is linked to high proliferation of plasma cells [7, 8, 9], and it has previously been shown that plasma cell proliferation is an independent predictor of progression from smouldering multiple myeloma to MM [10]. The plasma cell proliferative index has classically been determined by flow cytometry‐based assays and more recently by gene expression profiling of purified plasma cells [7, 11, 12]. A recent paper from the Multiple Myeloma Research Foundation (MMRF) CoMMpass study showed that patients with a high proliferation subtype, determined using the proliferative index established by Bergsagel et al. [12], had drastically reduced median overall survival of only 21 months [13]. Updated prognostic biomarkers that integrate proliferative status will clearly help identify patients with adverse outcome.

Such prognostic biomarkers may include circular RNAs (circRNAs), a class of non‐coding RNAs. Rather than by canonical linear splicing, circRNAs are formed by a back‐splicing reaction where an upstream 5′ splice site is linked to a downstream 3′ splice site. This closed‐loop structure makes circRNAs more stable than linear RNAs [14, 15, 16]. Together with their slow biogenesis rates, this causes circRNAs to accumulate in non‐proliferating cells while being diluted in fast‐proliferating cells [17, 18]. Several studies also find higher circRNA expression in normal tissue than in tumour tissue [19, 20, 21, 22, 23] and in mantle cell lymphoma, another B‐cell malignancy, circRNA expression is inversely correlated with cell proliferation [24].

Despite their prognostic potential in cancer, circRNAs remain remarkably understudied in MM, in part since circRNAs cannot be detected in traditional polyA‐enriched RNA sequencing datasets [25, 26], and the link between proliferation and circRNA levels in MM has not been explored. In particular, there is a lack of studies using deep sequencing, and patient cohorts are often heterogeneous, for example including both newly diagnosed patients and relapse patients [27, 28, 29]. Additionally, there is often a lack of consideration regarding the purity of the used patient material.

In this study, we carried out the most comprehensive study of circRNAs in MM to date by performing deep total RNA sequencing (RNA‐seq) on two MM cohorts: a Danish cohort consisting of whole BM samples from 45 MM patients and 13 HCs, and a Norwegian cohort consisting of CD138‐purified plasma cells from 43 MM patients. Both cohorts are well‐characterised, and all MM samples were taken at the time of diagnosis prior to treatment initiation. We comprehensively profiled the circRNA landscape and compared circRNA expression between whole BM and purified plasma cells. We show that in whole BM, previously published proliferation indexes [11, 12] correlated positively with overall survival, while in purified plasma cells, proliferation correlated negatively with overall survival. Global circRNA expression levels likely reflect proliferation, and expression of individual circRNAs in purified plasma cells has great prognostic potential.

## 2 Methods

### 2.1 Patient Samples

The Danish cohort of 45 whole BM MM samples and 13 HCs (whole BM material) has been previously described [30]. The HCs were slightly younger and therefore not perfectly age‐matched to the MM samples. The study was approved by the Central Denmark Region Committee on Health Research Ethics (M‐20100171). The Norwegian cohort consisted of CD138‐purified plasma cells from 43 MM patients from the MM biobank at St. Olavs Hospital, Norway (Biobank1). The study was approved by the Regional Committee for Medical and Health Research Ethics (REK 175311). The CD138‐positive cells were isolated using a RoboSep automated cell separator and the Human CD138 Positive Selection Kit (StemCell Technologies, Grenoble, France). All samples were from the time of diagnosis. An overview of patient characteristics can be found in Table 1.

**TABLE 1:** Characteristics of all patients.

	Whole BM MM samples (*n* = 45)	Whole BM HCs (*n* = 13)	CD138‐purified plasma cell MM samples (*n* = 43)
Sex, *n* (%)			
Female	20 (44)	6 (46)	18 (42)
Male	25 (56)	7 (54)	25 (58)
Age*, years			
Median (range)	70 (45–87)	59 (46–73)	69 (46–91)
R‐ISS/ISS**, *n* (%)			
1	7 (16)		8 (19)
2	33 (73)		12 (28)
3	3 (7)		15 (35)
Not available	2 (4)		8 (18)
Cytogenetic feature (FISH), detected (tested)			
1q21+	0 (0)		8 (18)
t(4;14)	3 (42)		7 (43)
t(11;14)	6 (42)		3 (18)
del(17p)	11 (42)		5 (42)
t(14;16)	1 (42)		1 (25)
Overall survival, months			
Median (range)	60 (0.3–140)		38 (2–88)

* Age at time of diagnosis.

** R‐ISS and ISS were used for staging of the Danish and Norwegian cohort, respectively.

### 2.2 RNA Extraction

To stabilise bone marrow RNA levels, whole BM aspirates were collected using PAXgene Bone Marrow RNA Tubes, and RNA was purified using the PAXgene Bone Marrow RNA Kit (Qiagen, Hilden, Germany) according to the manufacturer's instructions. RNA from purified plasma cell MM samples was extracted using the AllPrep DNA/RNA Mini Kit (Qiagen) according to the manufacturer's instructions. RNA from the MM cell line OPM‐2 was extracted using the AllPrep DNA/RNA/miRNA Universal kit (Qiagen) according to the manufacturer's instructions.

### 2.3 Library Preparation and Total RNA‐Seq

For the Danish cohort, 500 ng of total RNA from each sample was rRNA depleted and prepared using the KAPA RNA HyperPrep Kit with RiboErase (Roche, Mannheim, Germany). Libraries were quality assessed using a Tapestation (Agilent, Santa Clara, California, USA) and Qubit (Thermo Fisher Scientific, Waltham, MA) and were pooled and sequenced as 100 bp paired end reads using the Illumina NovaSeq 6000 (Illumina, San Diego, California, USA), including 1% PhiX as in‐lane control. The average sequencing depth was 122 M reads (range 53–176 M reads) (Figure S1A).

For the Norwegian cohort, 500 ng of total RNA from each sample was rRNA‐depleted and prepared using the SMARTer Stranded Total RNA Sample Prep Kit—HI mammalian (Takara, Kusatsu, Japan) with 12 PCR cycles for amplification. Libraries were quality assessed using the 2100 Bioanalyzer with the High Sensitivity DNA Kit (Agilent) and were pooled and sequenced as 100 bp paired end reads on the Illumina NovaSeq 6000 (Illumina), including 1% PhiX as in‐lane control. The average sequencing depth was 82 M reads (range 62–121 M reads) (Figure S1B).

For the experiments using the MM cell line OPM‐2, 100 ng of total RNA from each sample was rRNA depleted and prepared using the Illumina Stranded Total RNA Prep Ligation with Ribo‐Zero Plus kit (Illumina). Libraries were quality assessed using the 2100 Bioanalyzer with the High Sensitivity DNA Kit (Agilent) and were pooled and sequenced as 150 bp paired‐end reads on the Illumina NovaSeq 6000 (Illumina), including 1% PhiX as in‐lane control. The average sequencing depth was 58 M reads (range 47–74 M reads) (Figure S1C).

### 2.4 RNA‐Seq Data Analysis

Raw reads were quality filtered (Phred score below 20) and adapter trimmed using Trim Galore v0.6.6. For linear RNA analysis, reads were mapped against hg19/GRCh37 using STAR v2.7.7a. Mapped reads were quantified using FeatureCounts v.2.0.1 with gene annotations from Gencode version 37. Linear RNA expression was normalised using DESeq2 [31] v1.34.0. To correct for the variable level of immunoglobulin transcription between samples that introduce bias in the normalisation, plasma cell‐specific transcripts (including immunoglobulin elements) were excluded in the Transcript Per Million (TPM) normalisation of raw featureCounts counts used to calculate proliferative index values. Reads originating from leftover ribosomal RNA from the ribodepletion step were estimated using bbsplit (bbtools v37.62). CircRNA expression was quantified using CIRI2 v2.0.6 [32] and find_circ v1.2 [16]. CircRNA counts were normalised using DESeq2 using both circular and linear counts.

### 2.5 Proliferative Indexes

Three thoroughly validated gene expression‐based proliferative indexes from Bergsagel et al. [12], Shaughnessy et al. [11] and Hose et al. [7] were used to determine proliferation in the patient samples. Calculation of the indexes was performed by log_2_ (TPM) normalisation of FeatureCounts counts. The Bergsagel and Shaughnessy proliferative indexes contain 12 and 11 genes, respectively, which are associated with proliferation, while the Hose proliferative index contains 50 genes specific for plasma cell proliferation [7]. The Hose proliferative index was therefore not applied to the whole BM cohort.

### 2.6 RT‐qPCR

For all RT‐qPCR analyses, 500 ng RNA was used as input material. cDNA conversion was performed using the M‐MLV kit (Thermo Fisher Scientific) for the MM patient samples and the SuperScript II Reverse Transcriptase (Thermo Fisher Scientific) for OPM‐2 samples. cDNA was diluted 1:5 in nuclease‐free water and 4 μL was mixed with 1X SYBR Green PCR Master Mix (Applied Biosystems, Waltham, MA). All primer concentrations were 10 μM. PCR was performed on the Lightcycler 480 (Roche) using the following conditions: 95°C for 10 min, followed by 45 cycles of 95°C for 10 s, 60°C for 30 s and 72°C for 30 s. Primer sequences can be found in Table S1. For visualisation of RT‐qPCR data, the 2^−Δ*C*^_*t*_ values were plotted using the mean of *PUM1* and *SF3A1* for normalisation, as these housekeeping genes previously have been shown to be stable in MM [33].

### 2.7 Cell Culture

The MM cell line OPM‐2 was purchased from DSMZ and cultured in RPMI1640 media with 10% foetal bovine serum (FBS) and 1% penicillin‐streptomycin at 37°C and 5% CO_2_. Cell proliferation and viability were measured on the LUNA‐II Automated Cell Counter (Logos Bioscience, Anyang, South Korea) in biological triplicates for the sequenced samples. The optimal FBS concentration for the serum starvation experiments was determined to be 1.5%, ensuring that cells stopped proliferating while limiting cell death. The cell starvation assay was designed with a control sample of OPM‐2 cells growing in the same media with 10% FBS for 96 h. The serum starvation sample was grown for 96 h with 1.5% FBS. The early recovery sample was grown for 96 h with 1.5% FBS, and subsequently the media was changed to contain 10% FBS for an additional 72 h. The late recovery sample was grown like the early recovery sample but grew in media with 10% FBS for 144 h.

### 2.8 Statistical Analysis

Statistical analyses were performed in R version 4.4.1 (The R Foundation for Statistical Computing, Vienna, Austria) and Graphpad Prism version 10.1.0 (GraphPad Software, San Diego, CA). Differential expression analyses on total RNA‐seq data of whole BM samples were performed using DESeq2, where *p*‐values were adjusted for multiple testing. All other *p*‐values were unadjusted. Correlation analyses were performed using the Spearman method. Mann‐Whitney tests were used to compare proliferative index values and expression levels between groups; two‐sided *p*‐values < 0.05 were considered significant. The following R packages were used: plotPCA for principal component analysis (PCA), pheatmap for cluster analysis and survival and survminer for survival analyses of individual circRNAs with minprop set to 0.2. The function surv_cutpoint from the survminer package was used to determine the optimal cutpoint for expression of individual circRNAs.

## 3 Results

### 3.1 The circRNA Landscape in Whole BM of MM Patients

**FIGURE 1:**
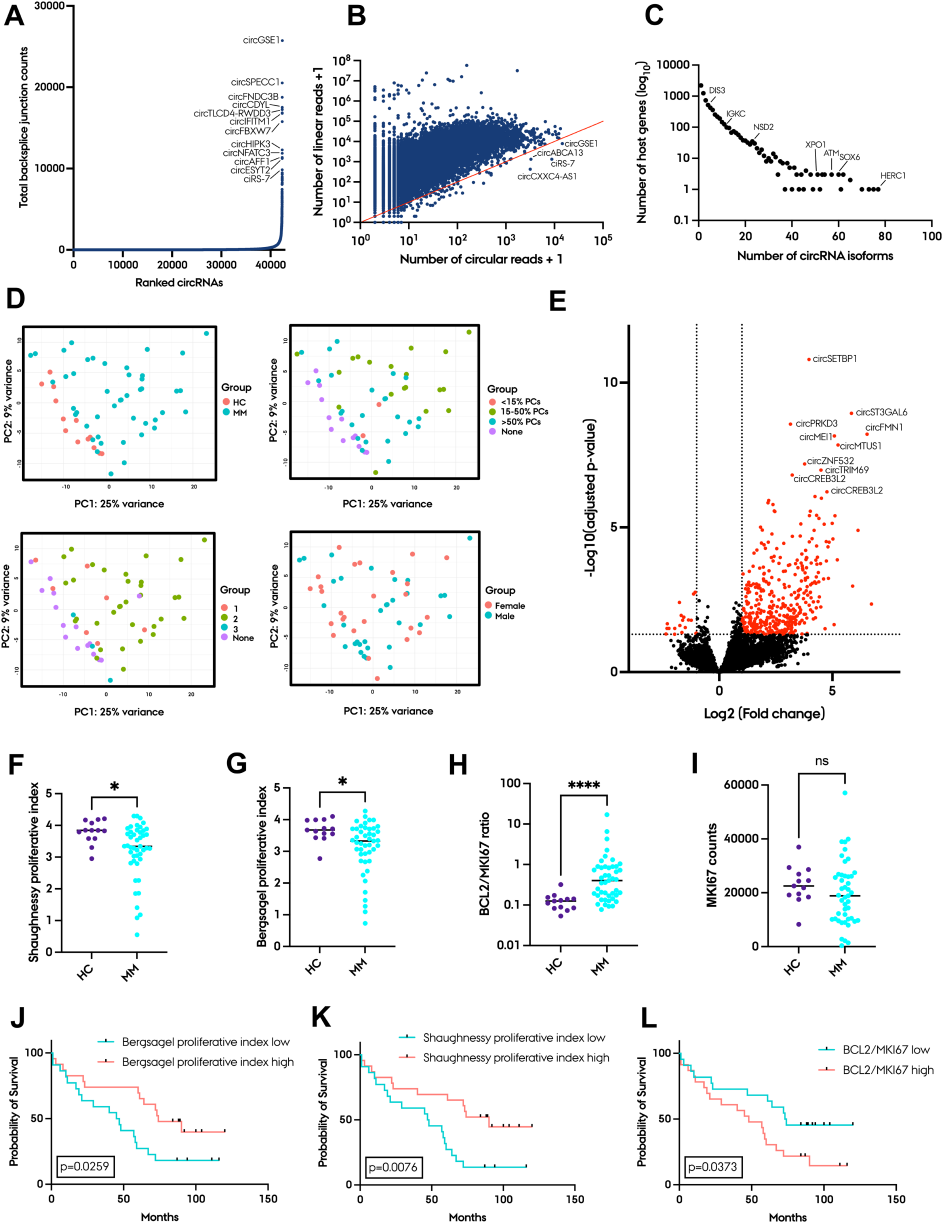
The circRNA landscape in whole BM of MM patients. (A) Number of circRNAs detected in whole BM samples (*n* = 58), with the most abundant circRNAs annotated. (B) Scatter plot comparing the number of circular reads and the corresponding linear reads for each circRNA (log_10_ scale). The red diagonal line separates circRNAs being expressed at higher levels than their cognate linear host gene (below the line). (C) Scatter plot of the total number of circRNA isoforms (x‐axis) per host gene (y‐axis, log_10_ scale). For instance, *DIS3* is one of 518 genes that produce 4 circRNA isoforms. (D) PCA of total circRNA expression with diagnosis (upper left), plasma cell percentage (upper right), R‐ISS (lower left) and sex (lower right) annotated. (E) Volcano plot comparing the log_2_ fold changes and significance (−log_10_[adjusted *p*‐value]) of individual differentially expressed circRNAs in diagnostic MM samples (*n* = 45) versus HCs (*n* = 13). The top 10 most differentially expressed circRNAs are annotated by circAtlas ID. Red dots are significantly differentially expressed circRNAs (log_2_ fold change > 1 and adjusted *p*‐value > 0.05). Right side shows upregulation of circRNAs in MM patients. (F, G) Shaughnessy and Bergsagel proliferative index values for HCs and MM samples. *p < 0.05. (H, I) *MKI67* levels and *BCL2/MKI67* ratios for HCs and MM samples. NS, not significant, ****p < 0.0001. (J–L) Kaplan‐Meier plots of Bergsagel and Shaughnessy proliferative indexes and *BCL2*/*MKI67* ratios (all with median cut‐off). Log‐rank *p*‐values between high and low groups are shown.

To thoroughly characterise the circRNA landscape in MM, we performed deep RNA‐seq of ribosomal RNA‐depleted total RNA from 45 MM whole BM samples collected at the time of diagnosis and 13 BM samples from HCs. No differences in sequencing depth or number of unique circRNAs were found between HC and MM samples (Figure S1A). We detected a total of 55,571 circRNAs supported by at least two back‐splice junction spanning reads by the CIRI2 pipeline across all samples. To ensure robust circRNA detection, we considered the overlap of circRNAs detected by CIRI2 and those either detected by the find_circ algorithm or present in at least one of the three main circRNA databases: circAtlas [34], CIRCpedia [35] and circBase [36]. This approach led to the identification of 42,379 circRNAs (Figure 1A). Only a small subset of the circRNAs (0.67%) were expressed at higher levels than their linear host gene (Figure 1B). Interestingly, *NSD2*, which is upregulated because of t(4;14) translocations in MM, produced 23 different circRNA isoforms. The gene that produced the most unique circRNA isoforms was *HERC1*. However, most genes produced only a single circRNA isoform (Figure 1C). PCA of total circRNA expression revealed clustering based on diagnosis, with greater variation observed for the MM samples than HCs. We also observed variation according to plasma cell percentage, while there was no gender‐specific clustering (Figure 1D).

### 3.2 Differential Expression of circRNAs and Proliferation Indexes Between MM Patients and HCs

Differential expression analysis by DESeq2 revealed a global upregulation of circRNAs in whole BM MM samples compared to HCs (Figure 1E). In total, 407 circRNAs were significantly upregulated and 19 circRNAs were significantly downregulated. When applying the Bergsagel and Shaughnessy proliferative indexes to determine the proliferation levels in the samples, it was observed that proliferation levels were significantly higher in HCs than in MM patients (Figure 1F,G). The *BCL2*/*MKI67* ratio, which is a measure of cell turnover, was lower in HCs than in MM patients (Figure 1H), indicating a higher cell turnover, however, *MKI67* levels alone were not significantly higher (Figure 1I). Since higher proliferation levels, assessed by gene expression, in purified plasma cells have been identified as a biomarker of poor survival [7, 8, 9, 13], we assessed the association between proliferation levels in whole BM samples and patient outcomes. Kaplan‐Meier analysis, performed on patients stratified into low and high proliferation groups based on the median levels, revealed that patients in the high proliferation group had a more favourable prognosis (Figure 1J,K). Likewise, a low *BCL2*/*MKI67* ratio was associated with longer survival (Figure 1L). When stratifying patients into low and high proliferation groups based on the median proliferative index levels of the HCs plus two standard deviations, the differences in overall survival were no longer significant (Figure S2A–C).

### 3.3 Patients With Low Proliferation Rates in Whole BM Samples Had Lower OS and High Overall circRNA Expression

**FIGURE 2:**
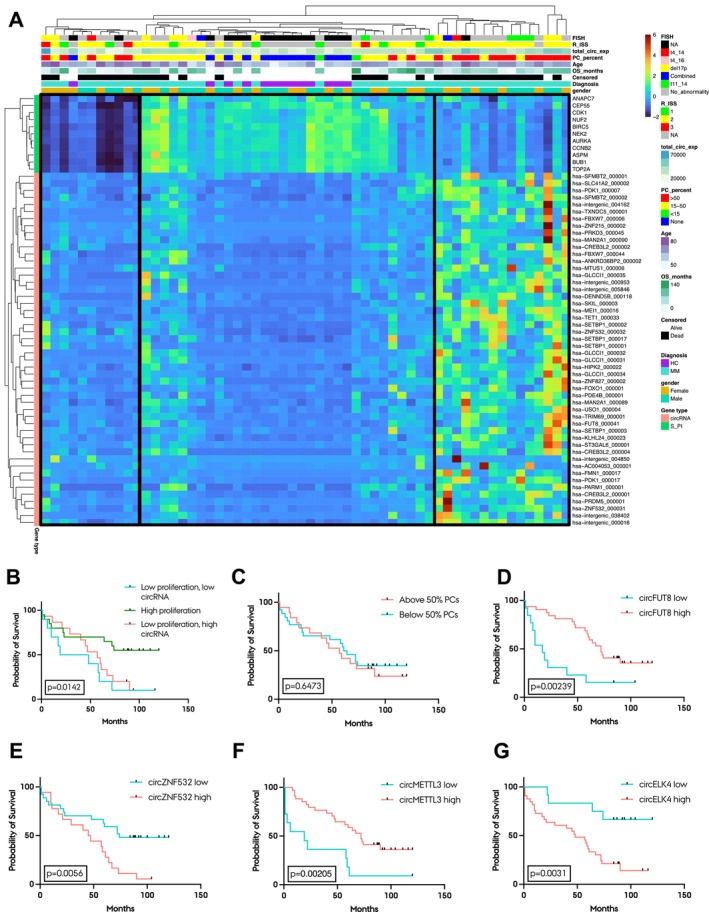
Patients with low proliferation rates in the BM have lower OS and high overall circRNA expression. (A) Heatmap of DESeq2 normalised z‐score transformed counts with hierarchical clustering of MM and HC samples and the top 50 most differentially expressed circRNAs (MM versus HC) (left side annotation, red), as well as the 11 genes present in the Shaughnessy proliferative index (S_PI) (left side annotation, green). Each column represents a patient, and each line represents a transcript (circRNA or S_PI gene). Translocation status (FISH), R‐ISS, total circRNA expression (total_circ_exp), plasma cell percentage (PC_percent), overall survival (OS_months), survival (Censored), diagnosis and gender are annotated. Three groups are highlighted: Left, patients with low proliferation and low circRNA expression; middle, patients with high proliferation; right, patients with low proliferation and high circRNA expression. FISH annotation: NA, not available; t4_14, t(4;14) translocation; t4_16, t(4;16) translocation; del17p, deletion of chr17p; Combined, two or more translocation groups present; t11_14, t(11;14) translocation; No_abnormality, none of the above‐mentioned abnormalities detected. (B) Kaplan‐Meier plot of the MM patients in the three groups indicated in (A). The Log‐rank *p*‐value between groups is shown. (C) Kaplan‐Meier plot of MM patients stratified into an above 50% plasma cell (PC) group or a below 50% PC group. The log‐rank *p*‐value between groups is shown. (D–G) Kaplan‐Meier plots based on expression of circFUT8 (D), circZNF532 (E), circMETTL3 (F) and circELK4 (G).

We investigated whether the MM BM samples with low proliferation levels corresponded to those with high circRNA expression and vice versa. Hierarchical clustering of the top 50 most differentially expressed circRNAs and the 11 genes present in the Shaughnessy proliferative index revealed a distinct cluster of 15 MM patients with low proliferation and high circRNA expression (Figure 2A). However, we also observed a cluster of 11 samples with low proliferation according to the Shaughnessy index, which displayed relatively low expression of the circRNAs. All except one of the HC samples were present in a third, larger cluster, which generally exhibited higher proliferation levels and lower circRNA expression. The survival of the MM patients in this cluster was significantly longer than the survival of the patients in the two other clusters (Figure 2B). There was no clustering observed according to age and gender, but the large cluster, containing most HC samples, had low levels of plasma cells compared to the other two clusters (Figure 2A). Thus, we investigated if the amount of plasma cells in the samples had an impact on patient survival; however, no difference in survival was found (Figure 2C). Next, we also investigated if expression levels of individual circRNAs were associated with survival. To this end, we performed Kaplan‐Meier analysis of the top 100 most differentially expressed circRNAs between MM samples and HCs and the top 100 most abundant circRNAs (with an overlap of six circRNAs). Of the top 100 differentially expressed circRNAs, 24 were significantly associated with survival (Table S2) and for 12 of these, high expression was associated with favourable outcome. The most significant circRNA with high expression being associated with better outcome was circFUT8 (Figure 2D), and the most significant circRNA with low expression being associated with better outcome was circZNF532 (Figure 2E). Of the top 100 most abundant circRNAs, 23 were significantly associated with survival (Table S3) and for 12 of these, high expression was associated with better outcome. The two most significant circRNAs were circMETTL3 (Figure 2F, high expression associated with better outcome) and circELK4 (Figure 2G, low expression associated with better outcome).

### 3.4 The circRNA Landscape in Purified Plasma Cells Is Markedly Distinct From That in Whole BM

**FIGURE 3:**
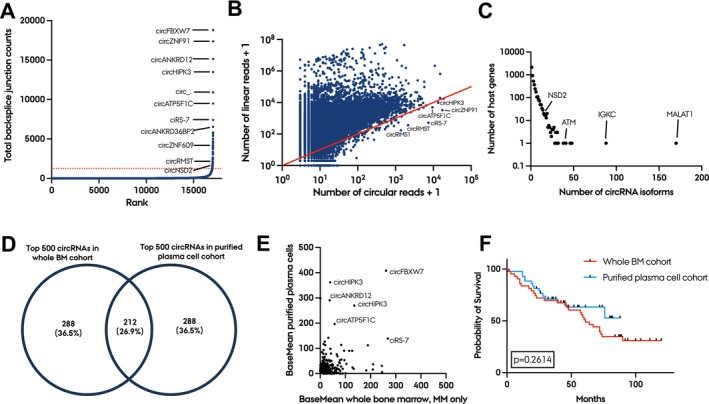
The circRNA landscape of purified plasma cells is markedly different from whole BM. (A) Number of circRNAs detected in purified plasma cell MM samples (*n* = 43), with the most abundant circRNAs annotated. (B) Scatter plot comparing the number of circular reads and the corresponding linear reads for each circRNA (log_10_ scale). The red diagonal line separates circRNAs being expressed at higher levels than their cognate linear host gene (below the line). (C) Scatter plot of the total number of circRNA isoforms (x‐axis) per host gene (y‐axis, log_10_ scale). (D) Venn diagram illustrating the overlap of the top 500 most abundant circRNAs in the whole BM cohort and purified plasma cell cohort (212 circRNAs). (E) Average expression (normalised counts from DESeq2) of the top 500 most abundant circRNAs in the whole BM cohort and the top 500 most abundant circRNAs in the purified plasma cell cohort. 49 circRNAs from the top 500 most abundant circRNAs in the whole BM cohort were not present in the purified plasma cell cohort, and 29 circRNAs from the top 500 most abundant circRNAs in the purified plasma cell cohort were not present in the whole BM cohort, leaving 710 circRNAs in the plot. (F) Kaplan‐Meier plot of survival in the whole BM cohort and the purified plasma cell cohort. The log‐rank *p*‐value between groups is shown.

Next, we examined the circRNA expression landscape in purified plasma cells from MM patients and compared it to that in whole BM. For this purpose, we studied a Norwegian patient cohort consisting of 51 diagnostic MM patient samples and applied a cutoff of at least 85% estimated purity, leaving 43 samples eligible for RNA‐seq. We further evaluated the purity *in silico* by the non‐B‐cell contamination index publicly available from the MMRF CoMMpass study and compared the values to the whole BM cohort (Figure S3A,B). All samples in the purified plasma cell cohort had values between 1.052 and 1.363, which indicates very high purity compared to the low purity subgroup described in the recent paper from the MMRF CoMMpass study [13]. We detected a total of 21,898 circRNAs supported by at least two back‐splice junction spanning reads by the CIRI2 pipeline across all samples. To ensure robust circRNA detection, we considered the overlap of circRNAs detected by CIRI2 and those either detected by the find_circ algorithm or present in at least one of the three main circRNA databases: circAtlas, CIRCpedia and circBase. This approach led to the identification of 18,482 circRNAs (Figure 3A), of which 1026 circRNAs (5.5%) were expressed at higher levels than their linear host genes (Figure 3B). The *NSD2* gene produced 15 different circRNA isoforms, while the *IGKC* and *MALAT1* genes produced 88 and 170 circRNA isoforms, respectively (Figure 3C). Within the top 500 most abundant circRNAs, 212 were also among the top 500 in the whole BM cohort (Figure 3D). The average expression levels varied greatly, indicating that some circRNAs are plasma cell‐specific while others are specific for other cell types in the BM (Figure 3E). There was no significant difference in survival between the two cohorts (Figure 3F).

### 3.5 Individual circRNAs Have Prognostic Potential in Purified Plasma Cell MM Samples

**FIGURE 4:**
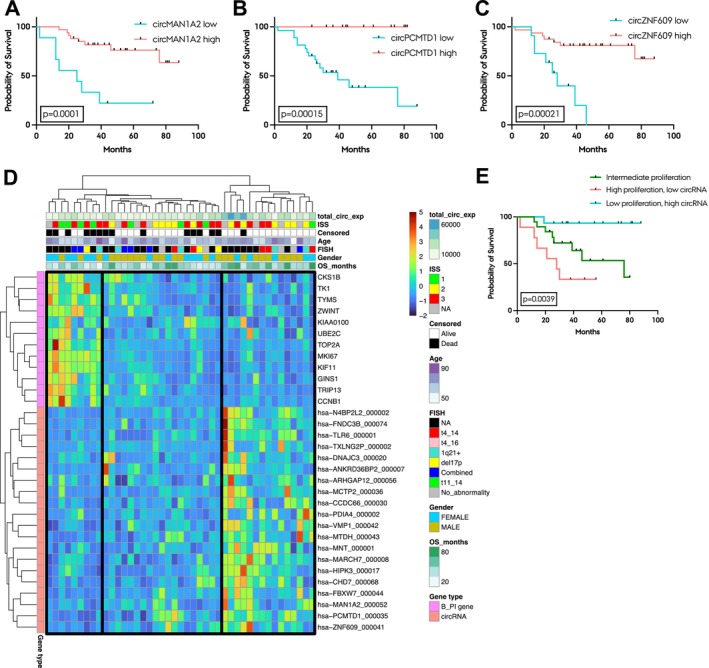
Individual circRNAs have prognostic potential in purified plasma cell MM samples. (A–C) Kaplan‐Meier plots of individual circRNA expression (circMAN1A2 (A), circPCMTD1 (B) and circZNF609 (C)). (D) Heatmap of DESeq2 normalised z‐score transformed counts with hierarchical clustering of MM samples and the top 20 circRNAs associated with survival (left side annotation, red) and the 12 genes present in the Bergsagel proliferative index (left side annotation, pink). Total circRNA expression (total_circ_exp), ISS, survival (Censored), age, translocation status (FISH), gender and overall survival (OS_months) are annotated. Three groups are highlighted: Left, patients with high proliferation and low circRNA expression; middle, patients with intermediate proliferation; right, patients with low proliferation and high circRNA expression. FISH annotation: NA, not available; t4_14, t(4;14) translocation; t4_16, t(4;16) translocation; del17p, deletion of chr17p; Combined, 2 or more translocation groups present; t11_14, t(11;14) translocation; No_abnormality, none of the above‐mentioned abnormalities detected. (E) Kaplan‐Meier plot of the three groups indicated in (D). The log‐rank *p*‐value between groups is shown.

Having observed a limited prognostic potential of circRNAs in the whole BM samples, we aimed to explore if individual circRNAs held more promise as prognostic biomarkers when evaluated in purified plasma cells. To this end, we performed Kaplan‐Meier analysis of the top 100 most abundant circRNAs to analyse if the expression levels of these circRNAs may be associated with survival. Of the top 100 most abundant circRNAs, 44 were significantly associated with survival. Strikingly, for 43 of these, high expression was associated with favourable outcome (Table S4). The most significant circRNAs with high expression being associated with better outcome were circMAN1A2, circPCMTD1 and circZNF609 (Figure 4A–C). Next, we performed hierarchical clustering of the top 20 most significant circRNAs positively correlated with survival and the 12 genes from the Bergsagel proliferative index. This analysis identified three distinct groups: one characterised by high proliferation and low circRNA expression, another by low proliferation and high circRNA expression, and a third ‘intermediate’ group displaying moderate levels of both proliferation and circRNA expression (Figure 4D). When stratifying patients into these three groups, we observed significantly different survival rates (Figure 4E). Patients in the high proliferation, low circRNA group had a hazard ratio of 13.75 (*p* = 0.0015) compared to patients in the low proliferation, high circRNA group. Finally, we investigated whether the top 20 significant circRNAs associated with survival in the purified plasma cell cohort had prognostic value in the whole BM patient cohort. Only three of these were found to be significantly associated with survival (Table S2).

### 3.6 circRNA Expression Correlates Negatively With Proliferation in Purified Plasma Cell MM Samples

**FIGURE 5:**
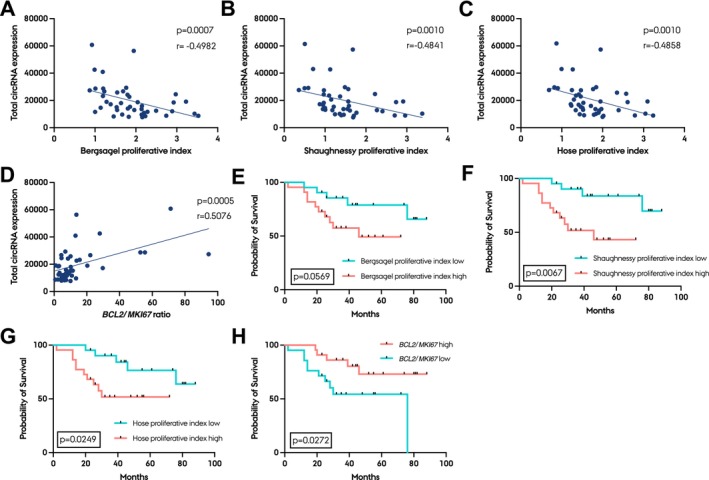
circRNA expression correlates negatively with proliferation in purified plasma cell MM samples. (A–D) Correlations of Bergsagel (A), Shaughnessy (B) and Hose (C) proliferative indexes and *BCL2*/*MKI67* ratio (D) with total circRNA expression. Spearman's correlation coefficient (r) and *p*‐value are shown for each correlation. E‐H Kaplan‐Meier plot of Bergsagel (E), Shaughnessy (F), Hose (G) proliferative index and *BCL2*/*MKI67* ratio (H) (all with median cut‐off). Log‐rank *p*‐values between high and low groups are shown.

Interestingly, a moderate negative correlation between all three proliferative indexes and total circRNA expression was observed in purified plasma cell samples (Figure 5A–C). In line with this, we observed a positive correlation between *BCL2*/*MKI67* ratio and total circRNA expression (Figure 5D). These RNA‐seq data could be confirmed by RT‐qPCR for *BCL2*, *MKI67* and ciRS‐7 (Figure S4A–C). Kaplan‐Meier analyses of the proliferative indexes as well as the *BCL2*/*MKI67* ratio showed the opposite pattern of survival compared to the analyses on the whole BM samples; low proliferation and high *BCL2*/*MKI67* were associated with better survival (Figure 5E–H). Patients in the high proliferation groups, according to the Bergsagel, Shaughnessy and Hose proliferative indexes, had a hazard ratio of 2.795 (*p* = 0.0569), 4.396 (*p* = 0.0067) and 3.427 (*p* = 0.0249), respectively, compared to patients in the low proliferation groups (Figure 5E–G).

### 3.7 circRNA Expression Reflects Proliferation in MM Cells

**FIGURE 6:**
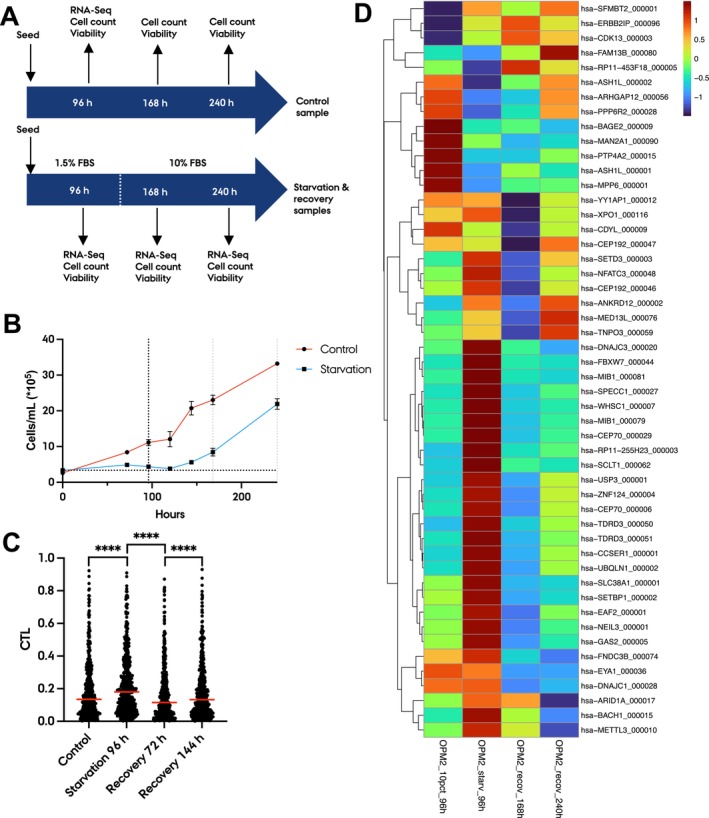
circRNA expression reflects proliferation in MM cells. (A) Illustration of the experimental setup for the starvation assay on the MM cell line OPM‐2. A sample grown in 10% FBS for 96 h was used as a control (top). After 96 h of starvation in 1.5% FBS, the starvation sample was harvested (bottom). After 96 h starvation and a subsequent 72 h (168 h timepoint) recovery in media with 10% FBS, the early recovery sample was harvested. The late recovery sample was harvested after 168 h of recovery (240 h timepoint). Cell count and viability were measured as indicated. (B) Cell counts for the OPM‐2 cells during the experiment. Samples used for sequencing were grown in biological triplicates and additional samples as duplicates or a single well. (C) Circ‐to‐linear ratios of all expressed circRNAs. ****p < 0.0001, Wilcoxon test. (D) Heatmap and unsupervised hierarchical clustering of circ‐to‐linear ratios (z‐scores) of the top 50 most abundant circRNAs in the control sample (OPM2_10pct_96h), starvation sample (OPM2_starv_96h), early recovery sample (OPM2_recov_168h) and late recovery sample (OPM2_recov_240h).

While individual circRNAs may function as tumour suppressors in certain cancers, it would be naïve to assume that all 43 circRNAs, which were positively correlating with survival in purified plasma cell MM samples, have tumour suppressor roles in MM. Instead, because of the correlation with proliferation, we hypothesised that our findings could be explained by the circRNAs being diluted in fast proliferating cells. To investigate this further, we set up a cell starvation assay using the MM cell line OPM‐2 (Figure 6A). After 96 h of serum starvation, the cells barely proliferated, but proliferation increased after 72 h of recovery in 10% FBS media (Figure 6B). After an additional 96 h of recovery, the cells started to become confluent and thus less proliferative again. The control cells with 10% FBS grew as expected. Viability was not significantly affected in the starvation sample compared to the control (Figure S5A). RT‐qPCR of *MKI67* (Figure S5B) and proliferative indexes (Figure S1) confirmed the observed proliferation levels. We performed total RNA‐seq on the control, starvation and recovery samples to investigate how proliferation levels may impact global circRNA levels. Since starvation causes widespread transcriptomic changes, we used circ‐to‐linear ratios when comparing circRNA expression between the samples. We found that circ‐to‐linear ratios significantly increased in starving cells compared to control cells (Figure 6C). In recovering cells, the total circRNA levels dropped, and then increased again after long recovery when cells became confluent. However, when focusing on the top 50 most abundant circRNAs, not all followed this pattern (Figure 6D).

## 4 Discussion

There is a need for better prognostic biomarkers in MM. CircRNAs are promising biomarker candidates [37], attributed in part to unique properties related to their covalently closed structures. However, circRNA studies in MM are scarce, as most sequencing studies, including the MMRF CoMMpass study, which is the largest sequencing study of MM patients [13], are based on a polyA enrichment during library preparation, precluding circRNA detection. We aimed to characterise the circRNA expression landscape in MM using RNA‐seq in two distinct cohorts of MM patients: one cohort of patients with whole BM samples and another cohort of patients with purified plasma cell samples. We detected more than 50,000 unique circRNAs and, to our knowledge, this is the most comprehensive sequencing study of circRNAs in MM to date. While most of the abundant circRNAs are well‐known from previous studies [37], only a few have been described in MM, including ciRS‐7 [38, 39] and circCDYL [40]. This observation underscores the need for more studies with deep total RNA‐seq to accurately capture and quantify circRNA expression in MM.

In whole BM samples, we show that circRNAs are more abundant in MM patients compared to HCs. The cellular composition of whole BM exhibits significant variation between HCs and MM patients. By definition, MM patients will have at least 10% clonal plasma cells present in their BM [4], while plasma cells usually comprise less than 2% of a healthy BM sample [6]. In the present study, MM patients had 10%–90% plasma cells in their BM. While we did not observe a correlation between the percentage of plasma cells and overall survival, contrary to previous research [41], we found that higher proliferation levels were associated with favourable prognosis. MM patients frequently present with anaemia [3], indicating that their BM is less functional with suppression of erythropoiesis and cells of the immune system [42, 43]. Our results indicate that the leftover non‐plasma cells, accounting for 10%–90% of the remaining cells, are less proliferative in MM patients, which could explain why we observe that higher proliferation levels are associated with a more favourable prognosis when measuring proliferation on whole BM. The higher circRNA expression in MM patients compared to HCs is, therefore, also in line with circRNAs accumulating in less proliferative cells [17, 18]. However, we also observed a cluster of patients with very low proliferation and low circRNA expression, indicating that proliferation cannot solely explain the circRNA expression levels. On the other hand, the purified plasma cell samples from MM patients revealed a cell‐type‐specific correlation between proliferation and circRNA expression, which suggests that it is crucial to consider the purity of MM samples in relation to proliferation levels. In these samples, high proliferation was associated with poor prognosis, which the circRNA levels also indicated. By manipulating cell proliferation rates, we showed that circRNAs tended to accumulate in non‐proliferating cells and become diluted in fast‐proliferating cells. These results indicate that circRNAs may not necessarily have a functional role as tumour suppressors simply because they are downregulated but are more likely to reflect proliferation levels [18]. Consequently, it is not within the scope of this paper to investigate potential functional roles of individual circRNAs. Rather, we demonstrate that circRNAs are very promising biomarkers in MM; in the present study, circRNAs provide additional prognostic value to the proliferative indexes, as evidenced by the HR being much larger in the low proliferation group when adding information on circRNA expression levels. Indeed, we found individual circRNAs being strongly associated with prognosis in purified plasma cell samples from MM patients. Since circRNAs have the highest prognostic value in purified plasma cells compared to whole BM, this, yet again, underscores the importance of sample purity.

In this study, only individual circRNAs were evaluated as prognostic biomarkers, which is a limitation of our study; it would also be highly relevant to evaluate a circRNA signature using machine learning. However, this would require an independent validation cohort, and it is evident that direct comparisons between the whole BM patient cohort and the purified plasma cell patient cohort are unsuitable.

In conclusion, circRNAs are abundant in MM and are promising prognostic biomarkers. CircRNAs are globally upregulated in MM patients compared to HCs in whole BM, where low proliferation and high circRNA levels are associated with a poor prognosis, reflecting the lack of healthy cells in the BM. In purified plasma cells, low proliferation and high circRNA levels are associated with a favourable prognosis, with individual circRNAs exhibiting great prognostic potential. Together, the findings presented here encourage further research to explore the prognostic value of circRNAs in MM.

## Author Contributions

**Theresa Jakobsen:** conceptualization (supporting), data curation (lead), formal analysis (lead), funding acquisition (supporting), investigation (supporting), methodology (supporting), project administration (supporting), software (lead), validation (equal), visualization (lead), writing – original draft (lead), writing – review and editing (equal). **Gro Grunnet Pløen:** data curation (supporting), formal analysis (supporting), investigation (supporting), methodology (supporting), writing – review and editing (supporting). **Alenka Djarmila Behsen:** data curation (supporting), methodology (supporting), resources (supporting), writing – review and editing (supporting). **Holger Jon Møller:** resources (equal), writing – review and editing (supporting). **Torben Plesner:** conceptualization (supporting), investigation (supporting), supervision (supporting), writing – review and editing (supporting). **Karen Dybkær:** conceptualization (supporting), investigation (supporting), supervision (supporting), writing – review and editing (supporting). **Morten Nørgaard Andersen:** conceptualization (supporting), data curation (supporting), investigation (supporting), methodology (supporting), writing – review and editing (supporting). **Kristine Misund:** conceptualization (supporting), data curation (supporting), investigation (supporting), methodology (supporting), resources (supporting), supervision (supporting), writing – review and editing (supporting). **Lasse Sommer Kristensen:** conceptualization (lead), formal analysis (supporting), funding acquisition (lead), investigation (lead), methodology (lead), project administration (lead), resources (supporting), supervision (lead), validation (equal), visualization (supporting), writing – review and editing (lead).

## Ethics Statement

The study was conducted according to the principles of the Declaration of Helsinki, and written informed consent was obtained from all patients participating in the study. The study was approved by the Central Denmark Region Committee on Health Research Ethics (M‐20100171) and the Regional Committee for Medical and Health Research Ethics (REK 175311).

## Conflicts of Interest

The authors declare no conflicts of interest.

## Supporting information


Data S1.

## Data Availability

Raw sequencing data and patient data are available upon reasonable request but will require ethical approval of the new research project and an agreement that allows data sharing between the data owner and the principal investigator/collaborators of the project and their institution(s).
