# Supplementary material for: The Prognostic Potential of circRNAs in Multiple Myeloma: Insights From Whole Bone Marrow and Purified Plasma Cells
Source: J Cell Mol Med. 2024 Nov 27;28(22):e70215. doi: 10.1111/jcmm.70215 (PMC11600292; doi:10.1111/jcmm.70215)
Supplement: Supplementary file 1 — Data S1. [file JCMM-28-e70215-s001.pdf]

Supplementary Table 1. RT-qPCR primer sequences

Name	Forward primer 5’-3’	Reverse primer 5’-3’
ciRS-7	ACGTCTCCAGTGTGCTGA	CTTGACACAGGTGCCATC
BCL2	ACGACTTCTCCCGCCGCTAC	CACCACCGTGGCAAAGCGT
MKI67	ATACGTGAACAGGAGCCAGC	CCTCACTCTCATCAGGGTCAGAAG
SF3A1	TCCATCCGTGAGAAGCAGAGC	TCTGGATCTCCTCCTCACCG
PUM1	CATGCCAGGTTATCCGGTGT	GCGCCTGCATTCACTACAAG

Supplementary Table 2. circRNAs significantly associated with survival from the top 100 differentially expressed circRNAs in whole BM patients compared to HCs.

circRNA	P-value	circAtlas ID	High expression is associated with more favorable prognosis?
circFUT8	0.00238	hsa-FUT8_000041	Yes
circGLCCI1	0.00309	hsa-GLCCI1_000032	Yes
circZNF532	0.0056	hsa-ZNF532_000031	No
circFMN1	0.00795	hsa-FMN1_000017	No
circSLC41A2	0.00864	hsa-SLC41A2_000002	Yes
circPDE4B	0.01053	hsa-PDE4B_000001	Yes
circATP6V0A1	0.01471	hsa-ATP6V0A1_000003	No
circFOXO1	0.01524	hsa-FOXO1_000001	Yes
circGLCCI1	0.01753	hsa-GLCCI1_000030	No
circCREB3L2	0.01857	hsa-CREB3L2_000001	No
circTXNDC11	0.0211	hsa-TXNDC11_000023	Yes
circMTUS1	0.02172	hsa-MTUS1_000006	No
circGLCCI1	0.02317	hsa-GLCCI1_000033	Yes
circPARM1	0.02359	hsa-PARM1_000001	No
circARHGEF12	0.02832	hsa-ARHGEF12_000006	Yes
circTULP4	0.02706	hsa-intergenic_000016	No
circDENND5B	0.0316	hsa-DENND5B_000118	No
circCADPS2	0.03537	hsa-CADPS2_000015	No
circPRKD3	0.04043	hsa-PRKD3_000045	Yes
circKLHL24	0.04244	hsa-KLHL24_000004	No
circCDK14	0.04589	hsa-CDK14_000074	Yes
circSKIL	0.04455	hsa-SKIL_000003	No
circTET1	0.04782	hsa-TET1_000033	Yes
circPVT1	0.04661	hsa-PVT1_000001	Yes

Supplementary Table 3. circRNAs significantly associated with survival from the 100 most abundant circRNAs in whole BM samples.

circRNA	P-value	circAtlas ID	High expression is associated with more favorable prognosis?

circMETTL3	0.00205	hsa-METTL3_000010	Yes
circESYT2	0.00315	hsa-ESYT2_000001	Yes
circELK4	0.00312	hsa-ELK4_000001	No
circRELL1	0.00354	hsa-RELL1_000007	Yes
circIFITM2	0.00657	hsa-IFITM2_000001	No
circCAMSAP1	0.0066	hsa-CAMSAP1_000014	Yes
circRBM33	0.00906	hsa-RBM33_000046	Yes
circEMB	0.01382	hsa-EMB_000006	Yes
circSLC38A1	0.0176	hsa-SLC38A1_000001	Yes
circCDYL	0.01822	hsa-CDYL_000009	No
circRHBDD1	0.02009	hsa-RHBDD1_000001	No
circABHD2	0.01963	hsa-ABHD2_000028	Yes
circZFAND6	0.02364	hsa-ZFAND6_000010	No
circMAN1A2	0.02827	hsa-MAN1A2_000052	Yes
circPTPN22	0.03073	hsa-PTPN22_000001	Yes
circHAGH	0.03384	hsa-HAGH_000001	No
circGPBP1L1	0.03508	hsa-GPBP1L1_000001	No
circPTPRA	0.03728	hsa-PTPRA_000059	No
circPRKD3	0.04043	hsa-PRKD3_000045	Yes
circDNAJC6	0.04132	hsa-DNAJC6_000026	No
circABCA13	0.0414	hsa-ABCA13_000007	Yes
circPHC3	0.04303	hsa-PHC3_000081	No
circCPSF6	0.04934	hsa-CPSF6_000028	No

Supplementary Table 4. circRNAs significantly associated with survival in purified plasma cell MM samples.

circRNA	p-value	circAtlas ID	High expression is associated with more favorable prognosis?	Significant in whole BM patient samples?
circMAN1A2	0.00014	hsa-MAN1A2_000052	Yes	Yes
circPCMTD1	0.00015	hsa-PCMTD1_000035	Yes	
circZNF609	0.00021	hsa-ZNF609_000041	Yes	
circFBXW7	0.0009	hsa-FBXW7_000044	Yes	
circTXLNGY	0.0013	hsa-TXLNG2P_000002	Yes	
circHIPK3	0.00258	hsa-HIPK3_000017	Yes	
circMNT	0.00273	hsa-MNT_000001	Yes	
circFNDC3B	0.0028	hsa-FNDC3B_000074	Yes	
circMARCHF7	0.00345	hsa-MARCH7_000008	Yes	
circN4BP2L2	0.00386	hsa-N4BP2L2_000002	Yes	Yes
circPDIA4	0.00506	hsa-PDIA4_000002	Yes	
circVMP1	0.00509	hsa-VMP1_000043	Yes	

circMCTP2	0.00543	hsa-MCTP2_000036	Yes	Yes
circCHD7	0.00632	hsa-CHD7_000068	Yes	
circTLR6	0.0071	hsa-TLR6_000001	Yes	
circARHGAP12	0.00819	hsa-ARHGAP12_000056	Yes	
circANKRD36BP2	0.00891	hsa-ANKRD36BP2_000007	Yes	
circDNAJC3	0.00899	hsa-DNAJC3_000020	Yes	
circMTDH	0.00955	hsa-MTDH_000043	Yes	
circCCDC66	0.00979	hsa-CCDC66_000030	Yes	
circMAN1A2	0.01011	hsa-MAN1A2_000051	Yes	
circCDYL	0.01041	hsa-CDYL_000009	Yes	
circRBM23	0.01163	hsa-RBM23_000002	Yes	
circANKRD36BP2	0.01173	hsa-ANKRD36BP2_000002	Yes	
circCEP70	0.01187	hsa-CEP70_000029	Yes	
circKPNA5	0.01299	hsa-KPNA5_000002	Yes	
circANKRD36BP2	0.01623	hsa-ANKRD36BP2_000008	Yes	
circMALAT1	0.01693	NA	Yes	
circVMP1	0.02394	hsa-VMP1_000042	Yes	
circRSRC1	0.02596	hsa-RSRC1_000025	Yes	
circRBM33	0.02619	hsa-RBM33_000046	Yes	
circLARP1B	0.0275	hsa-LARP1B_000002	Yes	
circPRKD3	0.02871	hsa-PRKD3_000045	Yes	
circRIMS1	0.02912	hsa-RIMS1_000156	Yes	
circSLC38A1	0.03137	hsa-SLC38A1_000001	No	
circSEC31A	0.03729	hsa-SEC31A_000008	Yes	
circZCCHC7	0.0392	hsa-ZCCHC7_000029	Yes	
circMED13L	0.04416	hsa-MED13L_000076	Yes	
circFNDC3B	0.04471	hsa-FNDC3B_000068	Yes	
circFAM13B	0.04509	hsa-FAM13B_000080	Yes	
circPNRC1	0.04531	hsa-PNRC1_000001	Yes	
circTUT7	0.04723	hsa-ZCCHC6_000001	Yes	
circZBTB44	0.04735	hsa-ZBTB44_000014	Yes	
circEXOC6B	0.04984	hsa-EXOC6B_000082	Yes	

Supplementary fig. 1 Total RNA sequencing results and circRNA output. A-C Total circRNA counts (left y axis, grey bar), number of unique circRNAs detected (left y axis, green bar), million (M) raw reads (right y axis, purple square) and percent rRNA reads (right y axis, orange dot) for each

sample in the whole BM cohort (**A**), purified plasma cell cohort (**B**) and OPM-2 cell line samples (**C**).

Supplementary fig. 2. A-C Kaplan-Meier plot of Bergsagel (**A**) and Shaughnessy (**B**) proliferative index and *BCL2*/*MKI67* ratio (**C**) of the 45 MM patients in the whole BM cohort. Cut-off based on median proliferative index value of HCs plus two standard deviations. Log-rank p-values between high and low groups are shown.

Supplementary fig. 3 *In silico* estimation of sample purity. A, B Non-B-cell contamination index values for each MM sample in the whole BM cohort (**A**) and purified plasma cell cohort (**B**). Line represents average value. The non-B-cell contamination index is publicly available from the MMRF CoMMpass study (https://github.com/tgen/MMRF_CoMMpass/tree/master/myeloma_purityCalculator_RNAseq).

Supplementary fig. 4. Confirmation of RNA-seq results with RT-qPCR. A-C RT-qPCR and normalized RNA-seq counts of the circRNA ciRS-7 (**A**), *BCL2* (**B**) and *MKI67* (**C**) on six patients with remaining material.

Supplementary fig. 5. Viability and proliferation of the MM cell line OPM-2. A Viability of OPM-2 cells at indicated time points. Error bars reflect biological replicates. **B** *MKI67* levels measured by RNA-seq (normalized counts) and RT-qPCR for OPM-2 samples. **C** Proliferative index values for OPM-2 samples. H_PI, Hose proliferative index; S_PI, Shaughnessy proliferative index; B_PI, Bergsagel proliferative index.

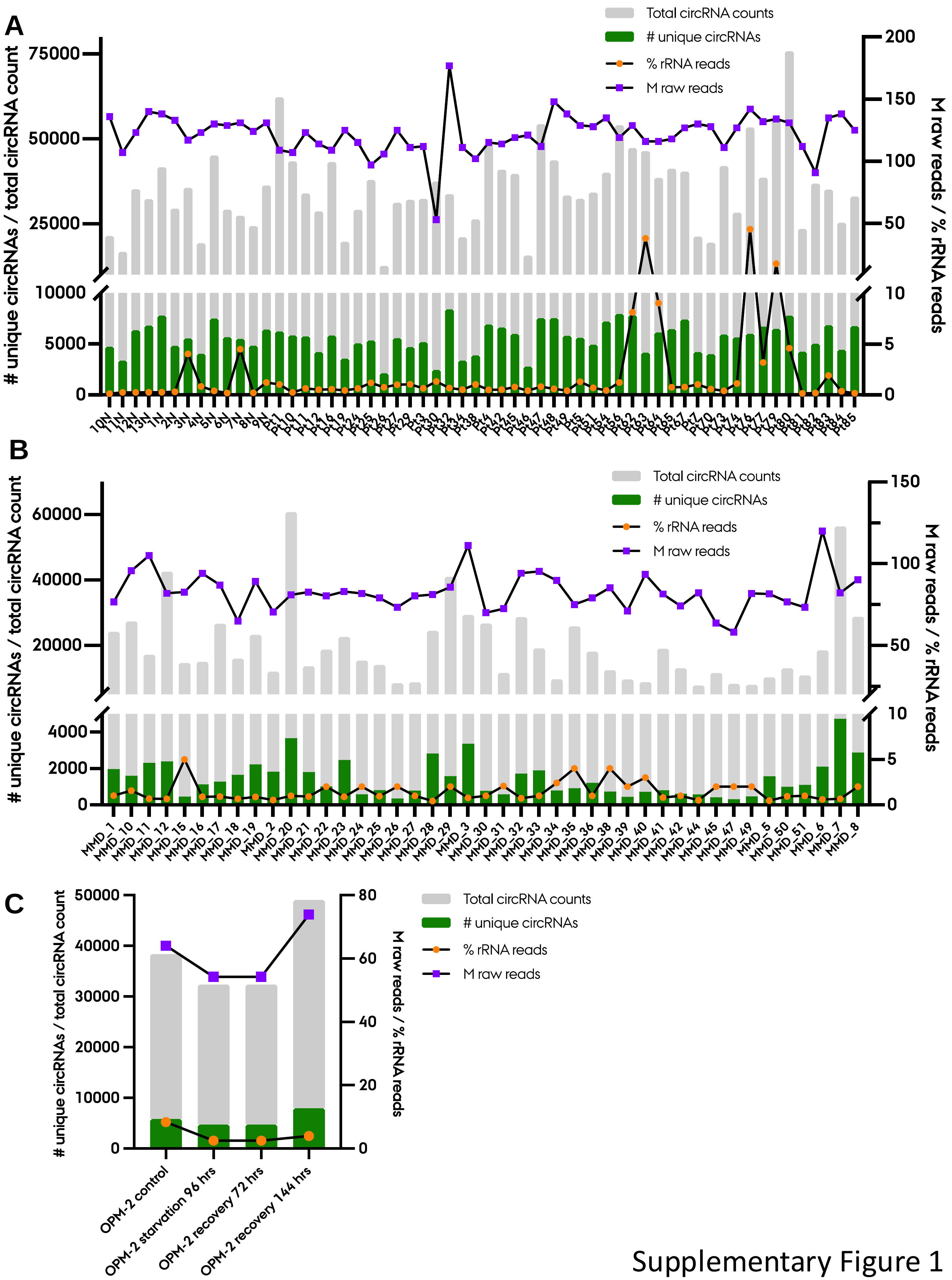

Supplementary Figure 1

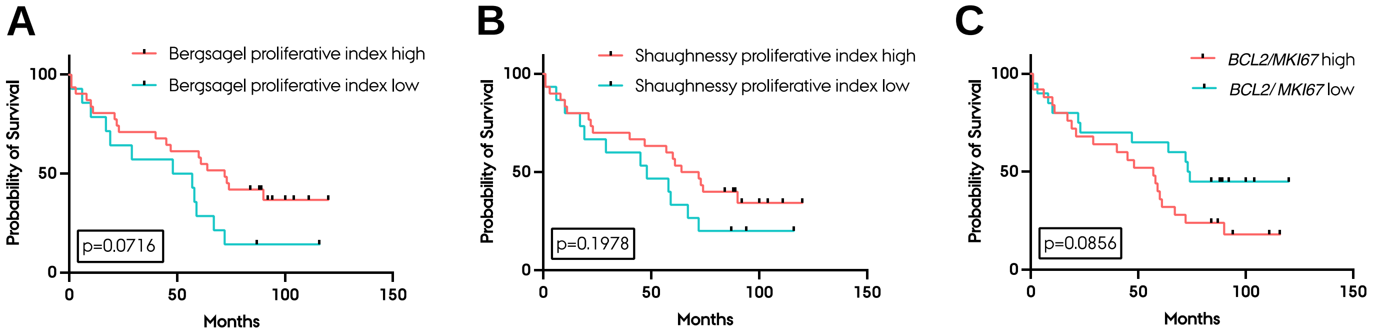

Supplementary Figure 2

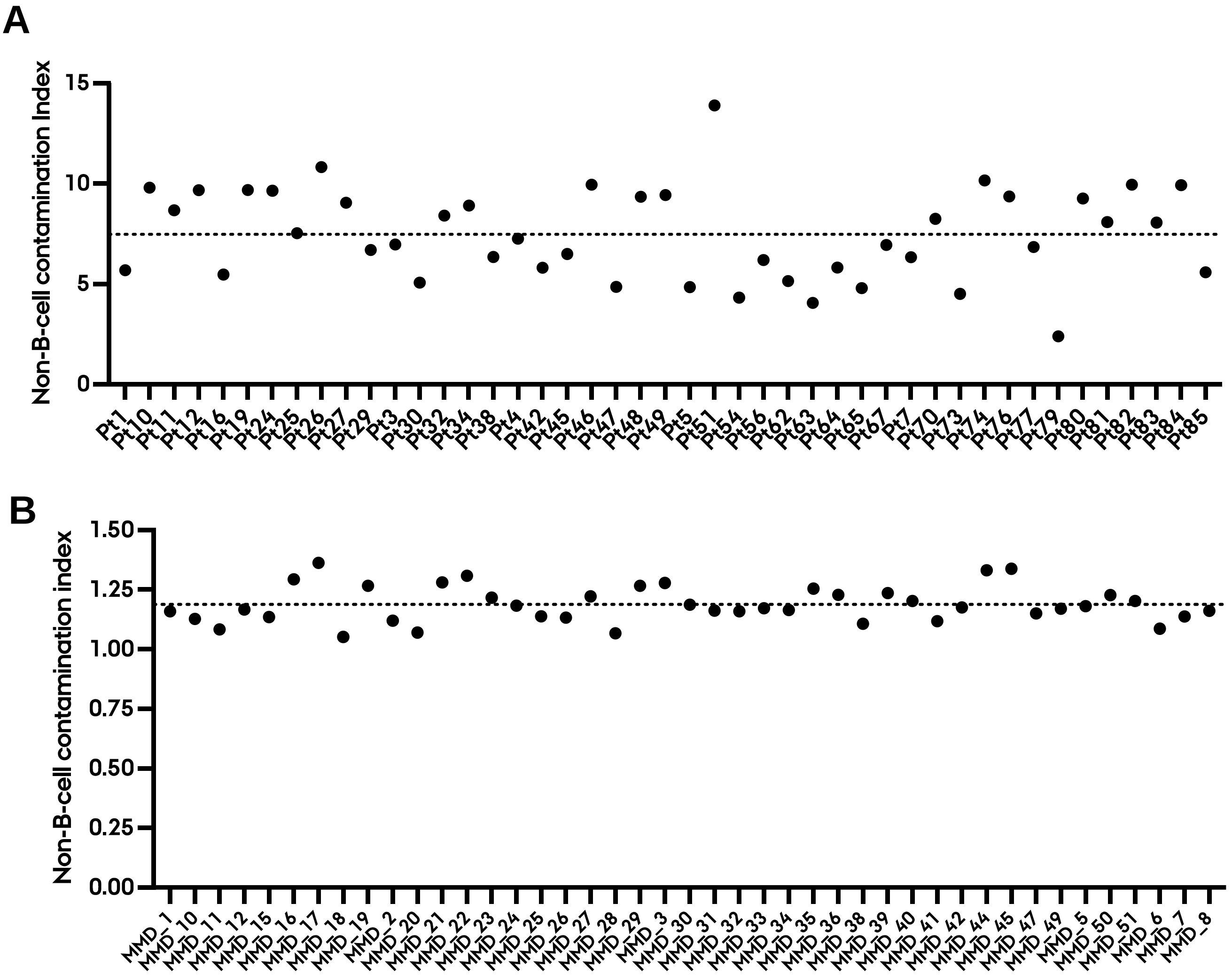

Supplementary Figure 3

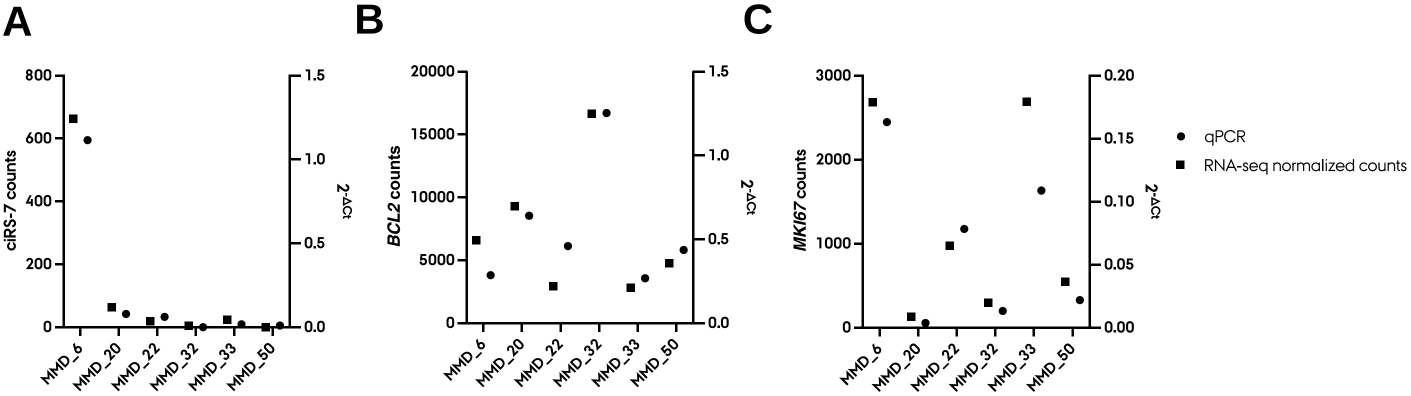

Supplementary Figure 4

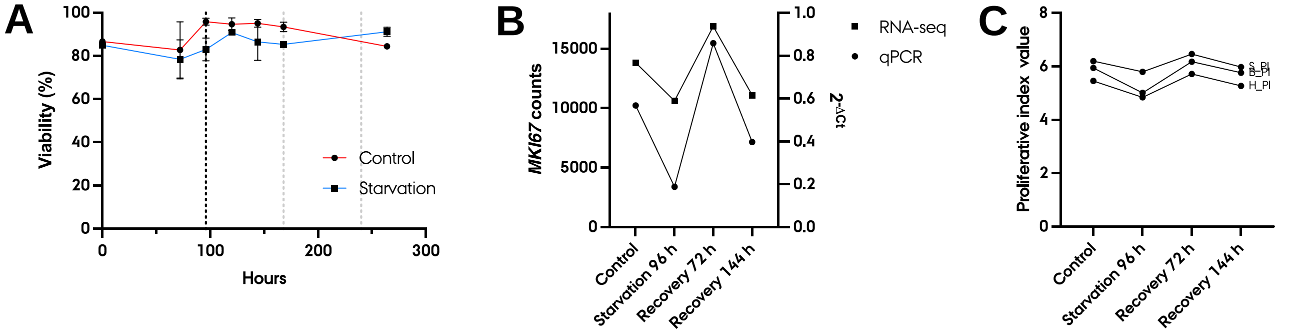

Supplementary Figure 5
